# The Hypoglycemic and Renal Protection Properties of Crocin *via* Oxidative Stress-Regulated NF-κB Signaling in db/db Mice

**DOI:** 10.3389/fphar.2020.00541

**Published:** 2020-04-30

**Authors:** Ye Qiu, Xue Jiang, Danping Liu, Zichun Deng, Weiwei Hu, Zhiping Li, Yuxin Li

**Affiliations:** ^1^National Engineering Lab for Druggable Gene and Protein Screening, Northeast Normal University, Changchun, China; ^2^School of Life Sciences, Jilin University, Changchun, China; ^3^School of Life Sciences, Northeast Normal University, Changchun, China

**Keywords:** crocin, diabetes, diabetic nephropathy, oxidative stress, inflammation, Nrf2 signaling

## Abstract

**Background:**

As the main ingredient of *Crocus sativus L*. (Iridaceae) extract, crocin- I (CR) has been reported to show various pharmacological activities. The aim of this study was to investigate the hypoglycemic and renal protection properties of CR in db/db mice.

**Methods:**

Eight-week-old db/db mice were treated with metformin (Met) (100 mg/kg) and CR (50 mg/kg) for eight weeks.

**Results:**

CR treatment showed hypoglycemic functions indicated by reduced bodyweight, food and water intake, plasma glucose, and serum levels of glycated hemoglobin A1c. Additionally, the CR group showed increased serum levels of insulin and pyruvate kinase, hypolipidemic functions indicated by the suppressed levels of total cholesterol and triglyceride, and enhanced levels of high-density lipoprotein cholesterol, which are also indicators of hypoglycemic functions. The renal protection function of CR was demonstrated by its protection of renal structures and its regulation of potential indicators of nephropathy. The anti-oxidation and anti-inflammation effects of CR were verified by enzyme-linked immunosorbent assay. In the kidneys of db/db mice, CR decreased the expression of phospho-IκBα and phospho-nuclear factor-κB (NF-κB), whereas it enhanced the expression of nuclear respiratory factor 2, manganese superoxide dismutase 1, heme oxygenase-1, and catalase.

**Conclusions:**

The anti-diabetic and anti-diabetic nephritic effects of CR were related to its modulation of oxidative stress-mediated NF-κB signaling.

## Introduction

Diabetes mellitus (DM), a metabolic and endocrine disease, affected over 114 million people in China in 2017. DM causes immune dysfunction and redox imbalance (Lebovitz, 1984; Xu et al., 2018). Specifically, hyperglycemia caused by gluconeogenesis or abnormal metabolism in DM patients is associated with dyslipidemia, insulin resistance, and many diabetic complications (Tangvarasittichai, 2015). The effects of abnormal metabolism on blood glucose and lipid levels are responsible for the development of diabetic nephropathy (DN), which is observed in nearly 40% of patients with type 1 DM (T1DM) and 20% of patients with type 2 DM (T2DM) (Yang et al., 2018). This leads to end-stage renal damage, recognized as the main cause of death in patients with diabetes (Yuan et al., 2018).

To understand the mechanism of DM, several hypotheses were proposed in previous studies. According to the “unified theory,” oxidative stress plays the crucial role during the development of DN and its complications (Chou and Tseng, 2017). Once oxidative stress occurs during hyperglycemia, reactive oxygen species (ROS) are generated, which regulate various cytokines involved in inflammation, glycometabolism, and lipometabolism (Wada and Makino, 2013; Sancar-Bas et al., 2015). Hyperglycemia, or increased blood glucose, is associated with hyperglycemia-induced mitochondrial dysfunction and endoplasmic reticulum stress, which promote ROS accumulation, which in turn promotes cellular damage and contributes to diabetic complications, such as DN (Fiorentino et al., 2013). During the development of DN, pro- and anti-inflammatory factors are produced by the innate cells of the kidney, increasing the inflammatory response (Banba et al., 2000).

In clinical practice, controlling glucose levels is the main therapy for DM. Patients with T1DM are often treated with insulin infusion, but patients can experience initial weight gain, hypoglycemia, and rashes at the injection site. In T2DM, the loss of efficacy over time is a major concern with the use of sulfonylureas, and the side effects of metformin (Met) can lead to gastrointestinal complaints in 30% of patients (Modi, 2007). Renal dialysis and transplantation will be performed after the patient develops end-stage of DN (Merrill, 1978; Hariharan et al., 1996). Based on the systematic research on the pathological mechanism of DN, natural agents for the effective control of the occurrence and development of DN have been searched for.

**Figure 1:**
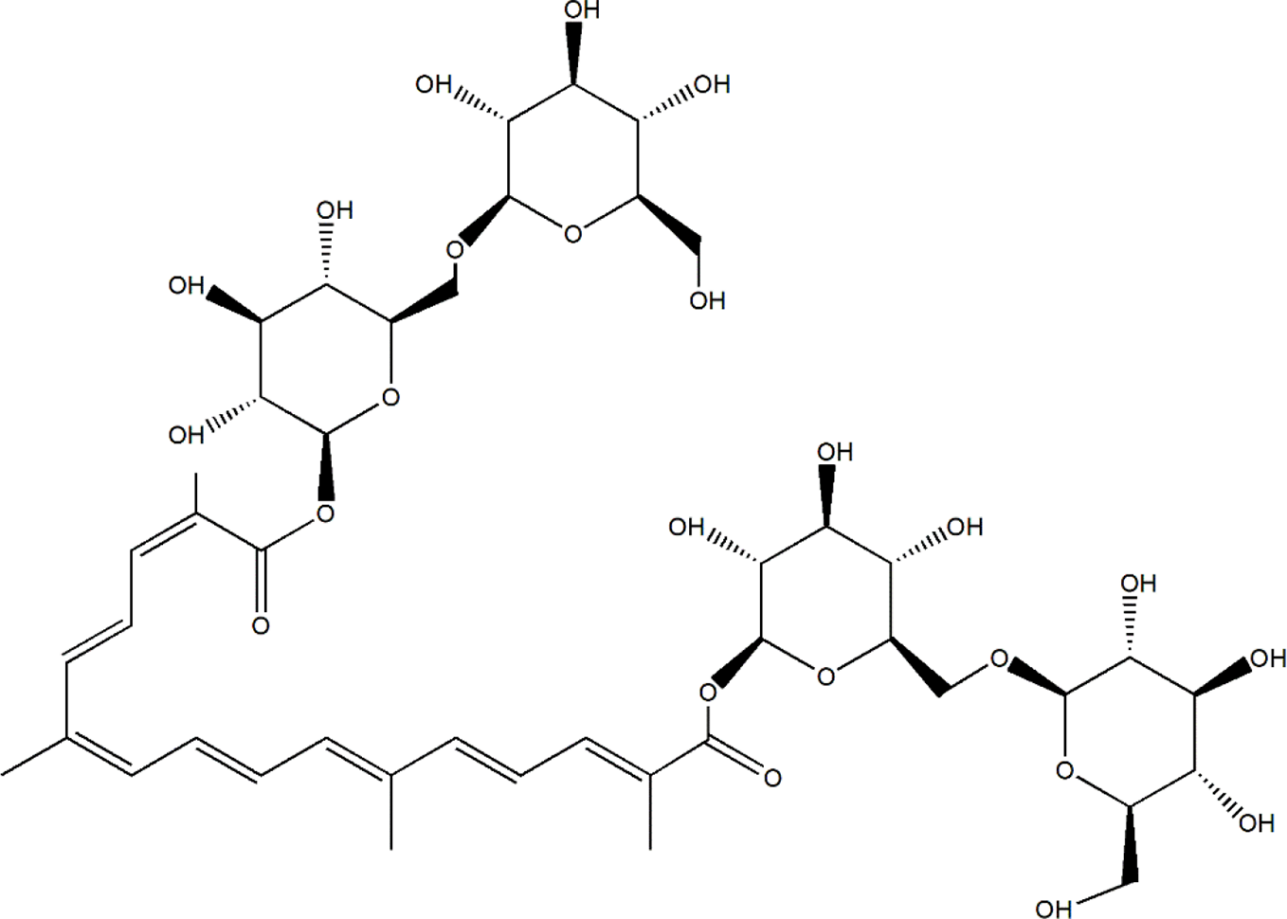
The molecular structure of CR.

Due to low toxicity and prospective effects, a resource library has been developed for searching for potential herbal compounds with anti-diabetic and anti-DN effects (Li et al., 2017). Saffron, the dried stigmas of *Crocus sativus L*. (Iridaceae), is one of the most expensive and valuable spices in the world, known as the red gold (Melnyk et al., 2010; Shahi et al., 2016). Saffron is cultivated in Iran, Mediterranean region such as Italy, Spain, and Greece, Northern Africa India (Kashmir) and some other countries in Europe and Asia (Yilmaz et al., 2010). Iran has been known as the largest world producer of saffron, accounting for nearly 90% production (Tabibian et al., 2020). Except for using as color and flavoring agent in the food industry, saffron always uses in traditional medicine due to its anti-convulsants, anti-depressants, anti-tumor, and effective in reducing blood glucose, fat, and cholesterol (Khazdair et al., 2015; Rodriguez-Ruiz et al., 2016). In a double-blind randomized clinical trial, 15 mg of saffron suppresses hyperglycemia and hyperlipidemia in type 2 diabetic patients (Aleali et al., 2019), and improves antioxidant indices in overweight/obese individuals with prediabetes (Karimi-Nazari et al., 2019). Crocin-I (Crocetin-di-beta-D-gentiobiosyl ester) (C_44_H_64_O_24_, molecular structure was shown as **Figure 1**), one type of crocin, has been known as the one of the pharmacological active constituents of saffron (Vahdati Hassani F et al., 2014), and its high performance liquid chromatography (HPLC) chromatogram of saffron extraction (50% methanol and 50% double distilled water) is shown in **Figure S1**. The anti-apoptotic, anti-depressant, anti-inflammatory, and anti-oxidative effects of Crocin-I (CR) has been reported (Ochiai et al., 2004; Karimi et al., 2010; Qi et al., 2013). CR (50 mg/kg) prevented retinal ischemia/reperfusion-induced apoptosis of retinal ganglion cells by activating the phosphatidylinositol 3 kinase/protein kinase B (PI3K/AKT) signaling pathway (Qi et al., 2013). In hyperlipidemic rats, CR quenched free radicals and ameliorated the damages of hyperlipidemia (Sheng et al., 2006), and displayed antihyperglycemic and antioxidant activity in streptozotocin-induced diabetic rats (Rajaei et al., 2013). However, no systematic study related to the hypoglycemic and renal protective effects of CR and its underlying mechanisms has been reported.

In this study, we demonstrated the hyperglycemic, hypolipidemic, and anti-DN effects of CR. Additionally, we observed that CR-mediated renal protection during hyperglycemia was related to the regulation of nuclear factor-κB (NF-κB) by nuclear factor erythroid-2–related factor 2 (Nrf2) signaling in db/db mice.

## Methods and Materials

### Mice and Treatments

The animal experiments were carried out in accordance with the Guiding Principles of Northeast Normal University Animal Ethics Committee (20180512). Thirty-six male C57BlKS/+Lepr^db^/JNju (db/db) mice (8 weeks old) and 12 db/m+ mice (8 weeks old) were purchased from Nanjing Biomedical Research Institute of Nanjing University, Nanjing, China (SCXK (Su) 2015–0001). All mice were housed in clear plastic cages (n = 4/cage) and maintained on a 12-h light/dark cycle (lights on 08:00–20:00 h) at 22 ± 1°C with water and food available *ad libitum*. The db/db mice were fed with a high-sucrose/high-fat diet (containing 60% fat, 20% protein, and 20% carbohydrate) and the db/m+ mice were fed with a normal diet (containing 10% fat, 22% protein, and 68% carbohydrate).

The db/db mice were divided into three groups and given 8 mL/kg of normal saline (vehicle-treated mice serving as model group, n=12), 100 mg/kg of Met (Shenzhen Haiwang Pharmaceutical Co. Ltd., Shenzhen, China) (positive control group) (n=12), or 50 mg/kg (selected according to the results of preliminary experiment) of CR (CAS: B21336, 98%, Yuanye Biotechnology Co. Ltd., China) dissolved in the physiological saline by gavage once per day for eight weeks. The 12 db/m+ mice were given 8 mL/kg of normal saline (control group) by gavage once per day for eight weeks. During the treatment period, the bodyweights and fasting blood glucose levels were measured every week. After 8-week administration, all mice were placed in metabolic cages separately, the amount of food and water intake within 24 h was recorded, and the 24-h urine output of each mouse was collected.

### Oral Glucose Tolerance Test

After the collection of the 24-h urine output, all mice underwent 4 h of fasting and were given 2 g/kg of glucose by gavage. The blood glucose levels were measured at 0 min (prior to the administration of glucose), and 30, 60, 120, and 240 min after glucose loading. According the following formula, the area under the blood glucose curve (AUC) was calculated:

Area under the curve (AUC) = (glycemia 0 min + glycemia 30 min) × 0.25 + (glycemia 30 min + glycemia 60 min) × 0.25 + (glycemia 60 min + glycemia 120 min) × 0.25 + (glycemia 120 min + glycemia 240 min) × 0.25.

### Histopathological Examination

After oral glucose tolerance test (OGTT), blood samples were withdrawn from each mouse from the caudal vein, and all mice were euthanized using carbon dioxide asphyxiation. Tissues, including liver and kidney, were collected. One part of each tissue was fixed in 10% formalin. The fixed liver and kidney were dehydrated using 70% to 100% ethyl alcohol, dealcoholized using xylene, embedded in paraffin, and cut into 5-µm-thick sections. The sections were deparaffinized in xylene and rehydrated using 100% to 70% ethyl alcohol in the reverse order. All samples were stained with hematoxylin and eosin (H&E) to analyze the pathological changes in liver and kidney under an inverted microscope CKX41 (Olympus, Tokyo, Japan).

### Biochemical Indexes Measurement

The levels of glycosylated hemoglobin A1 (GHbA1c, MM-0511M1), insulin (INS, MM-0579M1), pyruvic kinase (PK, MM-0592M1), total cholesterol (TC, MM-0632M1), triglyceride (TG, MM-0631M2), high-density lipid cholesterol (HDL-C, MM-44105M1), albumin (ALB, MM-44286M1), interleukin 1β (IL-1β, MM-0040M1), interleukin 2 (IL-2, MM-0701M1), interleukin 4 (IL-4, MM-0165M1), interleukin 10 (IL-10, MM-0176M1), matrix metalloproteinase-9 (MMP-9, MM-0048M1), granulocyte colony-stimulating factor (G-CSF, MM-0186M1), ROS (MM-43700M1), malondialdehyde (MDA, MM-0897M1), superoxide dismutase (SOD, MM-0389M1), catalase (CAT, MM-44125M1), and glutathione peroxidase (GSH-Px, MM-0758M1) in the serum; the levels of N-acetyl-β-d-glucosidase (NAG, MM-0539M1) and blood urea nitrogen (BUN, MM-0692M1) in the urine; and the levels of cAMP-dependent protein kinase (PKA, MM-43805M1) and 6-keto prostaglandin F 1α (6-Keto-PGF1α, MM-0264M1) in the kidneys were analyzed by enzyme-linked immunosorbent assay (ELISA) kits (Jiangsu Kete Bio-Technology Co. Ltd., Jiangsu, China) according to the manufacturer's instructions.

### Western Blotting

One part of kidney was homogenized with lysis buffer containing 98% RIPA buffer, 1.0% 50-mM phenylmethanesulfonyl fluoride (PMSF) and 1.0% protease inhibitor cocktail, which were all purchased from Sigma-Aldrich, St. Louis, Missouri, USA. Equal amounts of protein in the tissue (30 μg) was separated by 10% to 12% sodium dodecyl sulfate-polyacrylamide gel electrophoresis (SDS-PAGE) and transferred onto polyvinylidene difluoride (PVDF) membrane (Merck Millipore, Burlington, Massachusetts, USA). The membranes were blocked using 5% bovine serum albumin (BSA) diluted in Tris-buffer for 4 h at 4°C, and incubated with primary antibodies including Nrf2 (ab17355) (Abcam, Shanghai, China), chloramphenicol acetyl transferase (CAT) (bs-2302R) (Bioss, Beijing, China), heme oxygenase 1 (HO-1) (ab68477) (Abcam, Shanghai, China), superoxide dismutase 1 (SOD-1) (bs-10216R) (Bioss, Beijing, China), T-inhibitor of κB alpha (T-IκBα) (ab32518) (Abcam, Shanghai, China), total (T)-NF-κB (ab76302) (Abcam, Shanghai, China), phospho-NF-κB (p-NF-κB) (ab86299) (Abcam, Shanghai, China), phospho-inhibitor of κB alpha (P-IκBα) (Ser36) (ab12135) (Abcam, Shanghai, China), and glyceraldehyde-3-phosphate dehydrogenase (GAPDH) (E-AB-20059) (Elabscience, Wuhan, China) with the dilution of 1:3000 at 4°C overnight. The membranes were washed with Tris buffered saline Tween (TBS-T) buffer, and further incubated with horseradish peroxidase-conjugated goat anti-rabbit secondary antibodies (KTSM1322, 1:2000, Kangti, Shenzhen, China) for 4 h at 4°C. Enhanced chemiluminescence (ECL) detection (5200) (Tanon, Shanghai, China) was used to visualize the bands under the gel imaging system (UVP, California, USA). Image J Version 1.8.0 (National Institutes of Health, Bethesda, USA) was used to detect the optical density of bands.

### Statistical Analysis

Data were expressed as mean ± SEM. Differences were determined by a one-way analysis of variance (ANOVA) followed by post-hoc multiple comparisons (Holm-Sidak test) using SPSS 22.0 software (IBM Corporation, Armonk, New York, USA). Statistical significance was declared for *P* values of less than 0.05.

## Results

### The Hypoglycemic Activity of CR in db/db Mice

**Figure 2:**
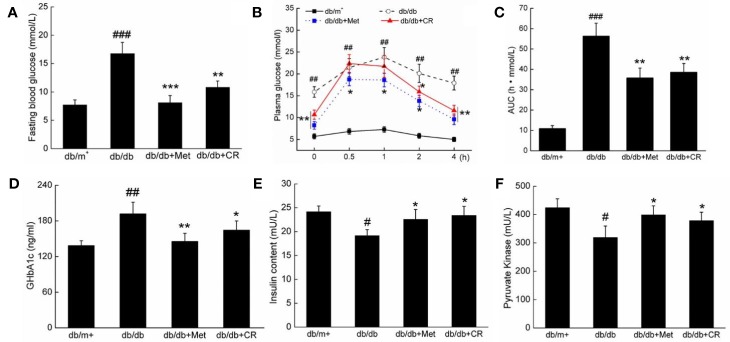
CR treatment affected the **(A)** fasting blood glucose, **(B)** oral glucose tolerance, **(C)** AUC, the serum levels of **(D)** GHbA1c, **(E)** insulin, and **(F)** PK in db/db mice compared to db/+ mice. The data were analyzed using a one-way ANOVA and expressed as mean ± SEM (n = 12). ^#^*P* < 0.05, ^##^*P* < 0.01, and ^###^*P* < 0.001 versus db/^+^ mice, *P < 0.05, **P < 0.01 and ***P < 0.001 versus non-treated db/db mice.

Compared with vehicle-treated db/db mice, Met and CR significantly reduced the fasting blood glucose levels (*P* < 0.05) (**Figure 2A**), and suppressed bodyweights after 8-week administration (*P* < 0.05) (**Table 1**). Enhanced food and water intake were observed in db/db mice, which were significantly suppressed by Met and CR (*P* < 0.01) (**Table 1**). Furthermore, hypertrophic liver and constrictive kidney in db/db mice were successfully reversed by CR (*P* < 0.05) (**Table 1**).

**Table 1:** The effect of Met and CR on the ratio of organ and body weight in db/db mice.

	Body weight (g)	Food intake (g/10 g)	Water intake (g/10 g)	Organ Indexes	
				Liver (%)	Kidney (%)
db/m+	26.4 ± 0.5	1.01 ± 0.12	3.02 ± 0.25	4.02 ± 0.58	1.52 ± 0.19
db/db	50.2 ± 1.4^###^	1.98 ± 0.11^###^	5.98 ± 0.66^###^	5.68 ± 0.22^##^	0.98 ± 0.08^##^
db/db+Met (100 mg/kg)	39.2 ± 2.1^**^	1.48 ± 0.10^**^	3.89 ± 0.43^***^	5.16 ± 0.56	1.24 ± 0.13*
db/db+CR (50 mg/kg)	42.6 ± 1.5^*^	1.45 ± 0.09^***^	4.02 ± 0.51^**^	4.56 ± 0.49*	1.31 ± 0.15*

The data were analyzed using a one-way ANOVA and expressed as means ± SEMs (n = 12). ^##^P < 0.01 and ^###^P < 0.001 versus db/m+ mice, *P < 0.05, **P < 0.01, and ***P < 0.001 versus non-treated db/db mice.

The dysfunction of glucose utilization and metabolism is observed in patients with diabetes due to insufficient INS secretion (Ostenson et al., 1993). In OGTT, CR significantly improved the blood glucose metabolism of db/db mice, demonstrated by suppressed blood glucose levels, especially 2 h after glucose administration (*P* < 0.05) (**Figure 2B**), and the area under the blood glucose curve (*P* < 0.01) (**Figure 2C**). Similar results of the OGTT were observed in Met-treated db/db mice (*P* < 0.05) (**Figures 2B, C**). Furthermore, compared with vehicle treated db/db mice, Met and CR showed beneficial effects on the levels of GHbA1c (**Figure 2D**), INS (**Figure 2E**), and PK (**Figure 2F**) in serum. CR reduced GHbA1c levels by 14.1% (*P* < 0.05) (**Figure 2D**), enhanced INS levels by 22.5% (*P* < 0.05) (**Figure 2E**), and enhanced PK levels by 18.5% (*P* < 0.05) (**Figure 2F**) in serum. In ITT, CR significantly improved the blood glucose metabolism of db/db mice (**Figure S2**).

### The Hypolipidemic Activity of CR in db/db Mice

**Figure 3:**
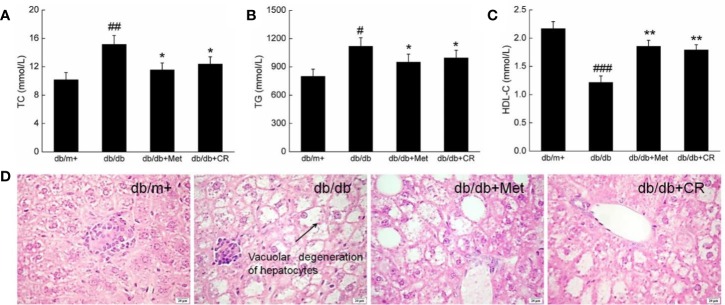
CR treatment affected the **(A)** TC, **(B)** TG, and **(C)** HDL-C in the serum of db/db mice. The data were analyzed using a one-way ANOVA and expressed as mean ± SEM (n = 12). ^#^*P* < 0.05, ^##^*P* < 0.01, and ^###^*P* < 0.001 versus db/+ mice, *P < 0.05 and **P < 0.01 versus non-treated db/db mice. **(D)** Histopathological analysis of the liver *via* H&E staining (scale bar: 20 μm; magnification: 400×). Arrow represents for vacuolar degeneration of hepatocytes of liver in db/db mice.

Hyperglycemia is responsible for pathological alternations in lipid metabolism, which cause obesity and other complications in patients with T2DM (Tung et al., 2018). Under normal circumstances, HDL-C promotes the metabolism of TC and TG by transferring from peripheral tissues to the liver (Song et al., 2019). Compared with db/m+ mice, high serum levels of TC, TG, and HDL-C were observed in db/db mice (*P* < 0.05) (**Figures 3A–C**). Met and CR significantly reduced the levels of TC (*P* < 0.05) (**Figure 3A**) and TG (*P* < 0.05) (**Figure 3B**) and enhanced the levels of HDL-C (*P* < 0.01) (**Figure 2C**) in the serum of db/db mice. Compared with db/m+ mice, fatty degeneration and lipid droplets were noted in db/db mice, which were significantly restored by Met and CR (**Figure 3D**).

### The Renal Protection of CR in db/db Mice Related to Anti-Inflammation

**Figure 4:**
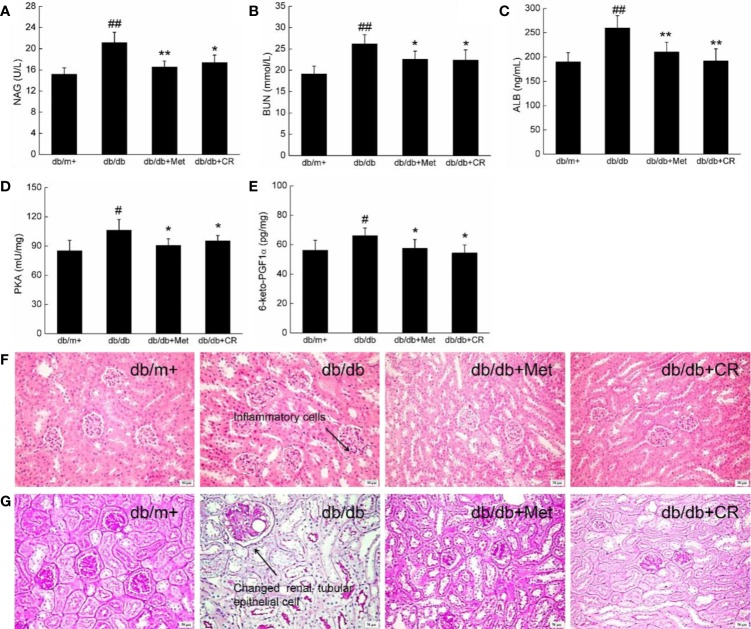
CR treatment affected the levels of **(A)** NAG, **(B)** BUN, **(C)** ALB, **(D)** PKA, and **(E)** 6-keto-PGF1α in the kidney of db/db mice. The data were analyzed using a one-way ANOVA and expressed as mean ± SEM (n = 12). ^#^*P* < 0.05 and ^##^*P* < 0.01 versus db/+ mice, *P < 0.05 and **P < 0.01 versus non-treated db/db mice. Histopathological analysis of kidney was shown by **(F)** H&E staining (scale bar, 50 μm; magnification, 200×) (arrow represents for inflammation cells of kidney in db/db mice) and **(G)** PAS staining (scale bar, 50 μm; magnification, 200×) (arrow represents for changed renal tubular epithelial cells of kidney in db/db mice).

Among the complications of T2DM, DN has been recognized as one of the most threatening. The biomarkers of DN, including urinary N-acetyl-beta-D-glucosaminidase (NAG) and blood urea nitrogen (BUN) (Lee and Lam, 2015), were enhanced in db/db mice (**Figures 4A, B**). In this study, the renal protection of CR during hyperglycemia was successfully demonstrated by the suppression of NAG (*P* < 0.05) (**Figure 4A**) and BUN (*P* < 0.05) (**Figure 4B**) in the urine, ALB in the serum (*P* < 0.01) (**Figure 4C**), and PKA (*P* < 0.05) (**Figure 4D**) and 6-keto-PGF1α (*P* < 0.05) (**Figure 4E**) in the kidneys of db/db mice after 8-week administration. In kidney tissues of db/db mice, inflammatory infiltration (**Figure 4F**) and pathological changes in renal tubular epithelial cells (**Figure 4G** and **Figure S3**) were noted, which were reduced after 8-week CR treatment, analyzed by H&E (**Figure 4F**) and periodic acid-Schiff (PAS) staining (**Figure 4G**). Besides, db/db mice exhibited more inflammatory cell infiltration than other groups (**Figure 4G**). Inflammatory cells hardly have cytoplasm, most of them are nucleus, which were stained as blue. Thus, a distinguished low background staining of db/db mice than others was observed.

Hyperglycemia down-regulates the expression and activity of MMPs in patients with DN, resulting in abnormal extracellular matrix (ECM) deposition (Xu et al., 2014). G-CSF prevents progression of early DN by mobilizing bone marrow cells to injured renal cells (So et al., 2013). Six cytokines in the serum of experimental mice related to pro- and anti-inflammation were detected *via* ELISA. Compared with vehicle-treated db/db mice, CR suppressed levels of IL-1β (*P* < 0.05) and IL-2 (*P* < 0.05) by 13.8% and 16.7%, and enhanced levels of IL-4 (*P* < 0.05), IL-10 (*P* < 0.05), MMP-9 (*P* < 0.05), and G-CSF (*P* < 0.05) by 16.4%, 28.2%, 26.7%, and 11.6%, respectively in the serum (**Table 2**). Met showed similar effects on reducing the levels of IL-1β and IL-2 and enhancing the levels of IL-4, IL-10, MMP-9, and G-CSF in the serum of db/db mice (*P* < 0.05) (**Table 2**).

**Table 2:** The effect of PHEA on the level of inflammatory cytokines in the serum of diabetic mice.

	db/m+	db/db	db/db+Met (100 mg/kg)	db/db+CR (50 mg/kg)
IL-1β (pg/mL)	80.2 ± 7.2	112.4 ± 10.2^##^	93.1 ± 8.9*	96.9 ± 10.1*
IL-2 (pg/mL)	19.8 ± 1.90	28.2 ± 2.23^##^	21.8 ± 1.88*	23.5 ± 2.26*
IL-4 (pg/mL)	126.4 ± 10.6	100.3 ± 9.2^#^	119.4 ± 10.4*	116.8 ± 11.2*
IL-10 (pg/mL)	41.6 ± 3.9	31.9 ± 3.3^##^	39.8 ± 2.2*	40.9 ± 4.4*
MMP-9 (ng/mL)	10.7 ± 1.1	7.5 ± 0.9^#^	9.2 ± 0.8*	9.5 ± 1.1*
G-CSF (pg/mL)	336.3 ± 32.1	286.3 ± 25.6^#^	329.5 ± 20.4*	319.5 ± 26.2*

The data were analyzed using a one-way ANOVA and expressed as means ± SEMs (n = 12). ^#^P < 0.05 and ^##^P < 0.01 versus db/m+ mice, *P < 0.05 versus non-treated db/db.

### The Anti-Oxidative Activities of CR in db/db Mice

In the normal state, the kidney generates a substantial amount of ROS, which is balanced by an extensive antioxidant system. However, in pathological states of hyperglycemia, the nitroso-oxidant balance shifts toward a pro-oxidant state that accelerates tissue and vascular injury, and leads to vascular dysfunction and kidney disease (Vasavada and Agarwal, 2005). Compared with db/m+ mice, the enhanced levels of ROS and MDA and the reduced levels of SOD, CAT, and GSH-Px in serum were noted in db/db mice (*P* < 0.05) (**Table 3**). In contrast, the 8-week CR treatment resulted in 24.2% and 26.9% reduction on the over-generated ROS (*P* < 0.05) and MDA (*P* < 0.05), and 25.8%, 36.1% and 35.1% enhancement on the levels of SOD (*P* < 0.05), CAT (*P* < 0.01) and GSH-Px (*P* < 0.05) in the serum of db/db mice (**Table 3**). Met showed similar effects as that of CR on the anti- and pro-oxidative stress factors in db/db mice (*P* < 0.05) (**Table 3**).

**Table 3:** The effect of CR on the level of oxidative-related cytokines in the serum of db/db mice.

	db/m+	db/db	db/db+Met (100 mg/kg)	db/db+CR (50 mg/kg)
ROS (U/mL)	329.3 ± 32.6	458.0 ± 42.3^##^	306.3 ± 34.5^**^	347.2 ± 37.9^*^
MDA (nmol/mL)	42.4 ± 5.2	68.8 ± 5.6^###^	49.2 ± 4.5^**^	50.3 ± 5.2^*^
SOD (U/mL)	120.8 ± 11.0	87.5 ± 9.5^##^	106.9 ± 9.9^*^	110.1 ± 9.3^*^
CAT (U/mL)	121.8 ± 11.5	52.1 ± 6.0^###^	78.6 ± 8.2^**^	70.9 ± 6.9^**^
GSH-Px (U/mL)	178.7 ± 14.5	143.8 ± 13.6^#^	171.2 ± 16.9^*^	178.9 ± 15.0^*^

The data were analyzed using a one-way ANOVA and expressed as means ± SEMs (n = 12). ^#^P < 0.05, ^##^P < 0.01, and ^###^P < 0.001 versus db/m+ mice, *P < 0.05 and **P < 0.01 versus non-treated db/db.

### Nrf2-Regulated NF-κB Activation Involved in CR-Mediated Renal Protection

**Figure 5:**
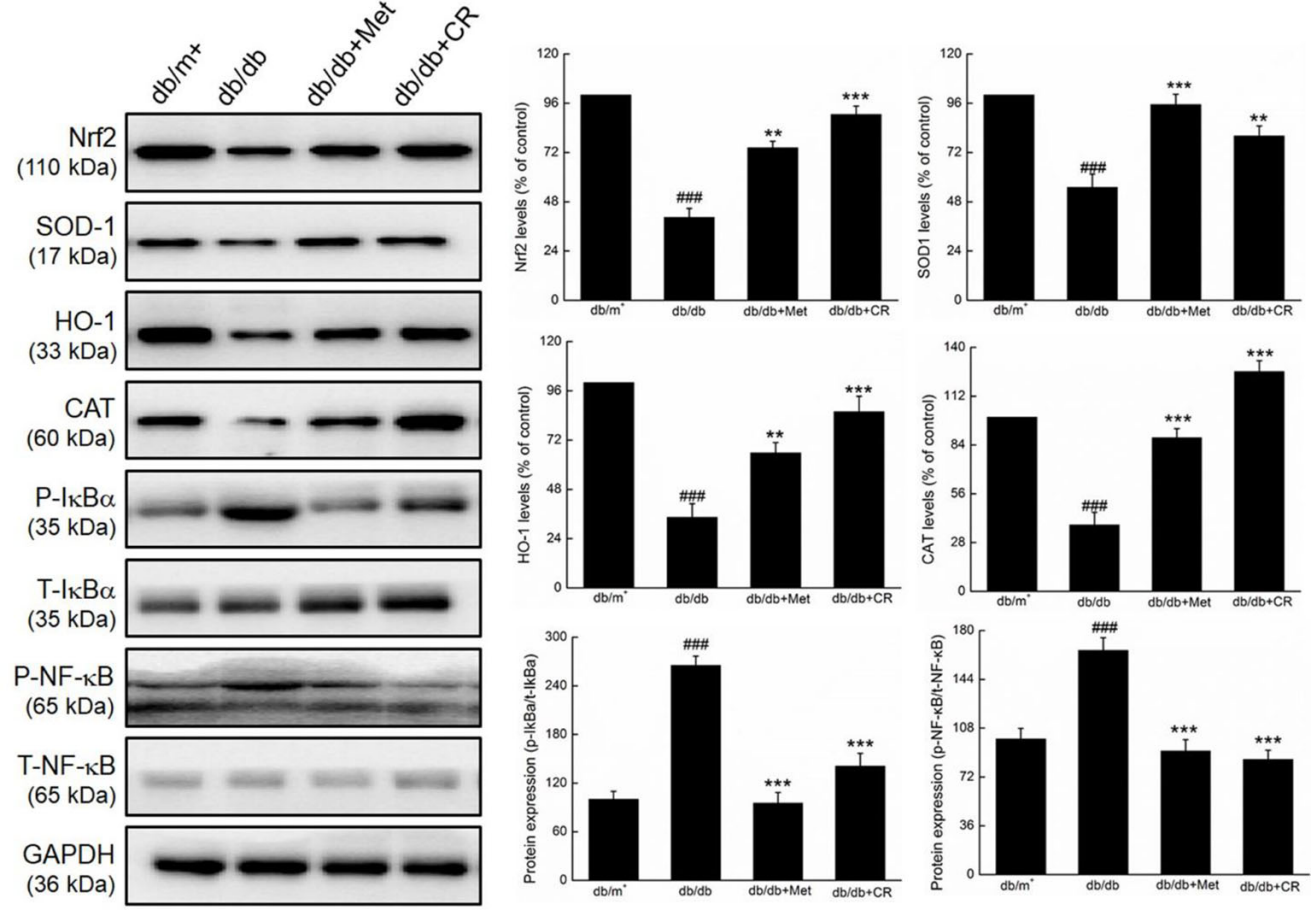
CR treatment regulated the expression of Nrf2, SOD1, HO-1, CAT, phospho-IκBα, and phospho-NF-κB in the kidney of db/db mice. Protein expression levels were normalized to the levels of GAPDH. The data were analyzed using a one-way ANOVA and expressed as mean ± SEM (n = 12). ^###^*P* < 0.001 versus db/+ mice, **P < 0.01 and ***P < 0.001 versus non-treated db/db mice.

Compared with vehicle-treated db/db mice, Met and CR significantly enhanced the expression levels of Nrf2 (*P* < 0.01), SOD-1 (*P* < 0.01), HO-1 (*P* < 0.01), and CAT (*P* < 0.001), and suppressed the phosphorylation of IκBα (*P* < 0.001) and NF-κB (*P* < 0.001) in the kidneys of db/db mice (**Figure 5**).

## Discussion

According to the previous studies, CR showed hypoglycemic and hypolipidemic effects in in STZ-induced type 2 diabetic rats (Shirali et al., 2013), and displayed hypoglycemic effects due to its antioxidative properties in streptozotocin-induced diabetes (Rajaei et al., 2013). Furthermore, CR reduced blood glucose level mainly *via* reduction of oxidative burden, modulation of apoptotic pathway, and attenuation of pancreatic inflammation in T1DM (Samaha et al., 2019). The most recent global estimates from the International Diabetes Federation (IDF) suggest that 449 million people had T2DM in 2017 (Zimmet et al., 2018). The db/db mouse, serving as the typical T2DM mouse model, exhibits insulin resistance (at around 2-week-age) and eventually develops hyperglycemia induced by β cell failure (4–8 weeks), which can accurately reflect the diabetes pathophysiology (Senturk et al., 2017). Till now, the renal protection of CR against DN in db/db mice has not been systemically reported.

In this study, we systemically reported the hypoglycemic, hypolipidemic, and renal protective activities of CR in db/db mice. Compared with currently used effective medicines (such as Met), CR exhibited a better effect on DN. CR significantly suppressed the hyper-levels of fasting blood glucose and GHbA1c and recovered the low levels of INS and PK. The enhanced gluconeogenesis combined with weakened glycolysis are noted in patients with diabetes (Ashcroft et al., 2017). Insufficient INS has been considered as a therapeutic target, which directly regulates glucose concentration (Insel et al., 1975). Insulin resistance is a primary pathogenesis of T2DM and can lead to related pathological changes such as hyperglycemia, hyperlipidemia, and hyperinsulinemia (Yang et al., 2019). PK is a kinase regulating glycolytic function. Glycolysis provides energy by the form of ATP in the fasted state. PK catalyzes the transfer of phosphate molecules from phosphoenolpyruvate to ADP and promote glycolysis (Ishwar et al., 2015).

The low levels of INS are responsible for dyslipidemia in patients with diabetes (Palazhy and Viswanathan, 2017). Combining with hyperglycemia, enhanced plasma lipid and lipoprotein levels are observed in diabetic patients, which may lead to nonalcoholic fatty liver (Chatrath et al., 2012). The accumulation of lipids, particularly TC and TG, in hyperglycemic patients caused by insufficient insulin can lead to diabetes-related complications (Palazhy and Viswanathan, 2017). In abnormal lipid metabolism, over generated plasma free fatty acids aggravate the impaired glucose metabolism in diabetes. CR strongly suppressed the levels of TC and TG and enhanced the levels of HDL-C in the serum of db/db mice.

Hyperglycemia causes the pathological elevation of glycated hemoglobin, which is responsible for the over-generation of ROS due to the suppression of the oxygen carrying capacity (Martin et al., 2010). This imbalance enhanced lipid peroxidation by the elevation of MDA in the plasma. MDA is the end product of lipid metabolism and is regulated by ROS. SOD, CAT, and GSH-Px have been recognized as first barriers for scavenging free radicals (Ouriel et al., 1985), which was strongly enhanced after 8-week CR administration. SOD, CAT, and GSH-Px scavenge ROS by dissimulating superoxide radicals, breaking down hydrogen peroxides and hydroperoxides into harmless molecules.

Combining with chaotic glucose and lipid metabolism during diabetes, the development of DN is often observed in DM patients. Oxidative stress causes the dysfunction of β-cells and helps to promote the production of pro-inflammatory mediators (Hasnain et al., 2016). CR restored the altered levels of NAG and BUN, which suggested its renal protection in db/db mice. In this study, CR suppressed the levels of IL-1β and IL-2, and enhanced the levels of MMP-9, G-CSF, IL-4, and IL-10 in the serum of db/db mice. Hyperglycemia and hyperlipidemia are responsible for the over-generation of pro-inflammatory cytokines, which may cause the glomerular infiltration of monocytes and macrophages, worsening the symptoms of DN (Arango Duque and Descoteaux, 2014). Due to the effects of IL-4 on priming naive T cells for a Th2 response, IL-4 plays a crucial role in interstitial fibrosis and inflammatory infiltration of the kidney during the development of DN (Ito et al., 2012). The progression of DN is usually simultaneous with severe inflammatory response and may develop into nephritides. The regulation of inflammatory factors such as IL-2, IL-10, and IL-1β is a method of kidney protection. Specifically, IL-10 regulates insulin resistance and hyperglycemia as an effective anti-inflammatory cytokine. The up-regulation of MMP-9 and G-CSF prevents the development of DN (Wang et al., 2019). Hyperglycemia brings about mitochondrial superoxide overproduction and intracellular ROS accumulation, which may lead to the activation of inflammatory pathways (Jiang et al., 2018).

According to a previous study, hyperglycemia is responsible for *NF-κB* overexpression, regulating NADPH-dependent oxidative stress and the renin-angiotensin system, which are involved in the development of DN (Kashihara et al., 2010). The activated IκBs activate NF-κB (Gomes et al., 2016), which transfers to the nucleus to control the genes encoding proinflammatory cytokines (Luo and Zheng, 2016). NF-κB activation is attenuated by diverse Nrf2 activators, such as phenethyl isothiocyanate (PEITC) and curcumin (CUR) (Jeong et al., 2004). Nrf2 is responsible for initiating the antioxidative response to ROS by controlling the expression of cellular phase-2 and antioxidant enzymes through activation of the antioxidant response elements (ARE). When the cells are being exposed to oxidative stress or xenobiosis, Nrf2 dissociates from Kelch-like ECH-associated protein (Keap1) and translocases into the nucleus, where it binds to ARE to induce gene expression of antioxidative stress enzymes, such as HO-1, SOD, and CAT to restore the imbalance of oxidative stress (Zhang et al., 2013). Evidence shows that natural products, such as extracts from *Auricularia cornea* (Mont.) Sacc., *Tuber melanosporum* Vitt., and *Cordyceps militaris* (L.) show renal protection by suppressing the activation of NF-κB. The anti-DN effects of CR are related to the modulation of NF-κB phosphorylation *via* the activation of Nrf2 signaling.

There is still limitation in this study. According to previous research, there is no significant difference in the risk of lactic acidosis and cardiovascular disease or elevated lactate levels in Met compared with other drugs (Salvatore et al., 2019). At the same time, new research has found that Met showed good effects in preventing metachronous colorectal adenoma or polyps, regulating intestinal flora and delaying aging (Higurashi et al., 2016; Zhao et al., 2019). Although in this study, in db/db mice, CR exhibited better regulatory effects on some of our experiments than those of Met, it does not mean CR is really better than Met. Our present data only support the further investigation on the possibility of CR as a candidate for diabetes and DN adjunct therapy.

In this study, CR was systemically investigated to show hypoglycemic, hypolipidemic, and renal protective activities in db/db mice, which are related to its anti-inflammation properties *via* regulating NF-κB through the activation of Nrf2 signaling.

## Data Availability Statement

All datasets generated for this study are included in the article/**Supplementary Material**.

## Ethics Statement

The animal study was reviewed and approved by The Guiding Principles of Northeast Normal University Animal Ethics Committee (20180512).

## Author Contributions

Conceptualization: YQ, XJ and ZD. Methodology: DL. Software: WH. Validation: YQ, XJ and ZD. Formal Analysis: YQ. Investigation: ZD. Resources: DL. Data Curation: WH. Writing—Original Draft Preparation: ZL. Writing—Review and Editing: YQ. Visualization: YL. Supervision: ZL. Project Administration: ZL. Funding Acquisition: ZL.

## Funding

This work was supported by Science Foundation in Jilin Province of P. R. China (Grant No. 20180101098JC) and the Special Projects of Cooperation between Jilin University and Jilin Province of P. R. China (SXGJSF2017-1).

## Conflict of Interest

The authors declare that the research was conducted in the absence of any commercial or financial relationships that could be construed as a potential conflict of interest.

The reviewer YF declared a shared affiliation, with no collaboration, with several of the authors, YQ, YL, DL, to the handling editor at the time of review.

## Abbreviations

6-keto-PGF1α, 6-Keto prostaglandin F 1α; ALB, albumin; ANOVA, analysis of variance; AUC, Area under the blood glucose curve; BSA, bovine serum albumin; BUN, blood urea nitrogen; CAT, catalase; CR, crocin; D-gal, D-galactose; DM, diabetes mellitus; DN, diabetic nephropathy; ELISA, Enzyme-linked immunosorbent assay; GAPDH, glyceraldehyde-3-phosphate dehydrogenase; GCSF, granulocyte colony-stimulating factor; GHbA1c, glycosylated hemoglobin A1; GSH-Px, glutathione peroxidase; H&E, hematoxylin-eosin staining; HDL-C, high-density lipid cholesterol; HO-1, heme oxygenase-1; INS, insulin; IκBα, inhibitor of κB alpha; MDA, malondialdehyde; Met, Metformin; MMP, mitochondrial membrane potential; NAG, N-acetyl-β-d-glucosidase; NF-κB, nuclear factor-κB; Nrf2, nuclear factor erythroid-2-related factor 2; OGTT, oral glucose tolerance test; PK, pyruvic kinase; PKA, cAMP-dependent protein kinase; PMSF, phenylmethanesulfonyl fluoride; PVDF, polyvinylidene difluoride; ROS, reactive oxygen species; SOD, superoxide dismutase; T1DM, type 1 DM; T2DM, type 2 DM; TC, total cholesterol; TG, triglyceride.
