# Supplementary material for: The Hypoglycemic and Renal Protection Properties of Crocin via Oxidative Stress-Regulated NF-κB Signaling in db/db Mice
Source: Front Pharmacol. 2020 Apr 30;11:541. doi: 10.3389/fphar.2020.00541 (PMC7212392; doi:10.3389/fphar.2020.00541)
Supplement: Supplementary file 1 [file DataSheet_1.docx]

**Method**

**The preparation of the stigma of *Crocus sativus L.* extraction**

The dried the stigma of *Crocus sativus L.* (Iridaceae) powder (Obtained from Changchun University of Chinese Medicine, Changchun, China) were extracted using ten-folds (w:w) 50% methanol solution dissolved in double distilled water at 30 ℃ for 30 min by ultrasonic twice.

**The HPLC analysis**

According to Chinese Pharmacopoeia 2015 version, the high performance liquid chromatography (HPLC) chromatogram of CR in the saffron extraction was analyzed. Shimadzu chromatograph equipped with C18 reverse phase column (250 mm × 4.6 mm, 5 μm) (E2912829, Liaoning, China) under the ultraviolet detector a was performed in this study. The conditions of HPLC as follows. The mobile phase was 45% methanol : 55% ultrapure water, the flow rate was 1.0 mL/min, the injection volume was 10 μL, the detected wavelength was 440 nm and the column temperature was 25 ℃.


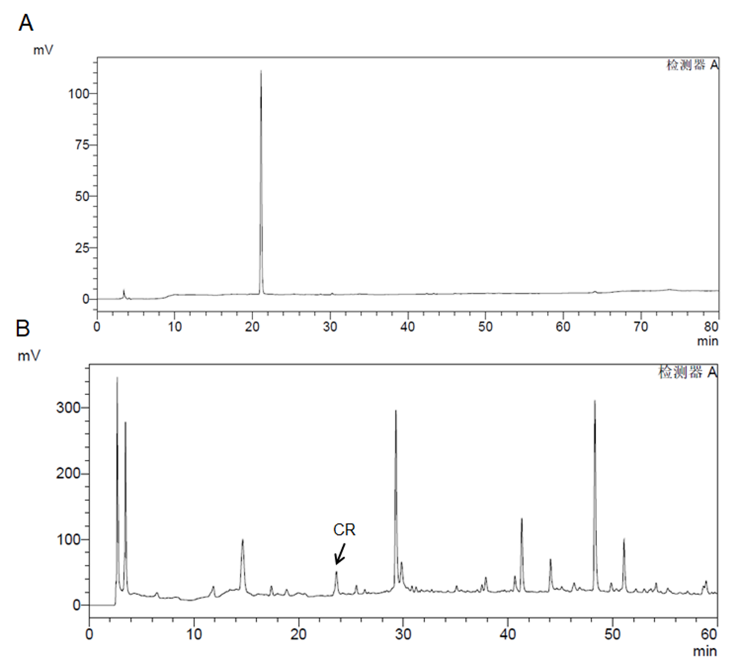


**Figure S1.** HPLC chromatograms of (**A**) the standard of CR and (**B**) CR in the saffron extraction.

**

**


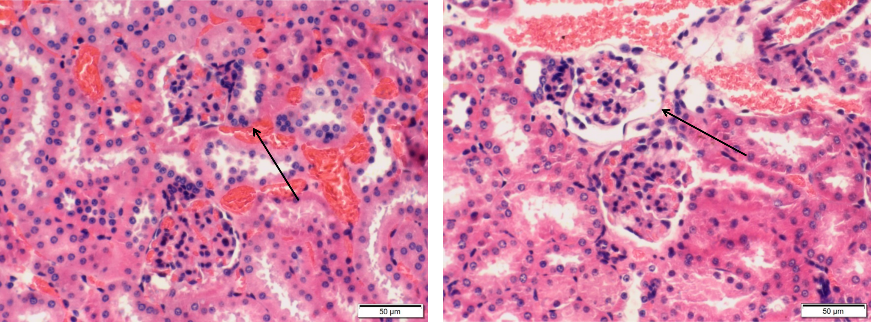
**Figure S2.** CR treatment affected the oral insulin tolerance in db/db mice compared to db/m+ mice. The data were analyzed using a one-way ANOVA and expressed as mean ± SEM (n = 12). ## P < 0.01 and ### P < 0.001 versus db/m+ mice, * P < 0.05 versus non-treated db/db mice.

**Figure S3.** Histopathological analysis of kidney in db/db mice was shown by PAS staining (scale bar: 50 μm; magnification: 200×) (Arrow represents for changed renal tubular epithelial cells of kidney in db/db mice).
